# Acute and sub-chronic oral toxicity study of purple sweet potato (*Ipomoea batatas* [L.] Lam) yogurt in mice (*Mus musculus*)

**DOI:** 10.14202/vetworld.2022.789-796

**Published:** 2022-03-31

**Authors:** Astrid Feinisa Khairani, Yunisa Pamela, Nandina Oktavia, Achadiyani Achadiyani, M. Yusuf Adipraja, Prita Yasri Zhafira, Widad Aghnia Shalannandia, Nur Atik

**Affiliations:** 1 Department of Biomedical Sciences, Division of Cell Biology, Faculty of Medicine, Universitas Padjadjaran, West Java, Indonesia; 2 Department of Biomedical Sciences, Division of Biochemistry and Biology Molecular, Faculty of Medicine, Universitas Padjadjaran, West Java, Indonesia; 3 Department of Biomedical Sciences, Division of Anatomy, Faculty of Medicine, Universitas Padjadjaran, West Java, Indonesia; 4 Undergraduate Program, Faculty of Medicine, Universitas Padjadjaran, West Java, Indonesia

**Keywords:** acute oral toxicity, anthocyanin, purple sweet potato, sub-chronic oral toxicity, yogurt

## Abstract

**Background and Aim:**

Food safety is an important aspect to be evaluated in preventing any potentially harmful side effects of food product such as yogurt. The purple sweet potato yogurt product was developed to combine the benefits of probiotic activities in yogurt and the bioactive effects of anthocyanin in purple sweet potato. This study was performed to investigate acute and sub-chronic oral toxicity of purple sweet potato yogurt (PSPY) in mice.

**Materials and Methods:**

Acute oral toxicity was evaluated by a 14-day observation for any clinical sign of toxicity on fifteen female balb/c mice following a single dosage of PSPY (nil, 2 or 5 g/kg body weight). The sub-chronic oral toxicity study was conducted by feeding PSPY to four groups of mice with the dose of 0, 12, 20, and 40 g/kg body weight for 28 days, and another group of mice receiving 40 g/kg body weight purple sweet potato for 14 days longer to observe any delayed toxicity effect. Body weight and clinical signs of toxicity were observed daily. Liver and kidney macroscopy and relative organ weight, liver histology, liver enzyme, and hematology profile analyses were done at the end of the study.

**Results:**

There were no signs of toxicity observed from the acute toxicity study and no abnormality in body weight, relative organ weight, and gross organ examination. In the sub-chronic toxicity study, there were no clinical signs of toxicity, no significant differences in body weight, relative liver weight, liver enzymes, hematology profile, or abnormality in gross and histological examination of the liver.

**Conclusion:**

This study shows that oral administration of PSPY in mice up to 5 g/kg body weight did not result in acute toxicity, while the dosage up to 40 g/kg body weight did not lead to sub-chronic toxicity.

## Introduction

With increasing popularity of foods that provide unbalanced nutrition, such as junk food and soft drinks, there is increasing evidence that eating habits affect one’s health. The previous study reported that increased consumption of such fast foods increased the risk of dyslipidemia, obesity, and cardiovascular disease [1]. In 2016, the World Health Organization estimated that about 39% of adults aged 18 years were overweight, and about 13% were obese in 2016. This obesity prevalence tripled from 1975 to 2016 [2]. Such a phenomenon drives the food sector to develop foods that provide excellent nutrition, such as producing functional foods [3]. Functional food is natural or processed food containing biologically active compounds. Within non-toxic amounts, these bioactive compounds are clinically proven and documented to provide health benefits for the prevention, management, and treatment of chronic diseases [4].

Purple sweet potato (*Ipomoea batatas* [L.] Lam) is a potential raw material for functional food. Aside from being a highly nutritious food source, it contains a high level of anthocyanin that provides antidyslipidemia, antioxidant, cardiovascular protection, and neuroprotection effects [5,6]. Purple sweet potato was used as an enrichment or prebiotic agent of yogurt to produce the purple sweet potato yogurt (PSPY) product. The product was developed to combine the benefits of probiotic activities in yogurt and the bioactive effects of anthocyanin contained in purple sweet potato.

There is limited data available regarding toxicity of purple sweet potato, specifically when it is combined with yogurt. However, a study of anthocyanin in grapes using mouse model reported that anthocyanin from grape skin extract did not show any toxic effects at the dosage of 25 mg/kg [7]. On the other hand, another study demonstrates that probiotics in yogurt led to several side effects in susceptible individuals such as systemic infection, deleterious metabolic activities, and excessive immune stimulation [8].

Hence, this study aims to evaluate the toxicity of PSPY by conducting acute and sub-chronic oral toxicity studies in mice.

## Materials and Methods

### Ethical approval

The use of animals for this research had been reviewed and approved by the Health Research Ethics Committee of Universitas Padjadjaran, Indonesia, registered as No.1430/UN6.KEP/EC/2019 for acute oral toxicity study and No.964/U.N.6KEP/EC/2019 for sub-chronic oral toxicity study.

### Study period and location

The study was conducted from April to September 2019 in Laboratories of Biomedical Sciences, Faculty of Medicine, Universitas Padjadjaran, Bandung, West Java, Indonesia.

### Bacterial starter culture

Lactic acid bacteria strains used in this study were *Lactobacillus bulgaricus* ATCC 11842*, Lactobacillus acidophilus* ATCC 4356, and *Bifidobacterium longum*; all were provided and confirmed to be used in this study by the Department of Biomedical Sciences, Faculty of Medicine, Universitas Padjadjaran. Bacterial cultures were grown on De Man, Rogosa and Sharpe agar for 24 h at 37°C. Each bacteria strain was inoculated in 100 ml commercial low fat pasteurized milk (PT Ultrajaya Milk Industry, Indonesia) and then incubated in 5% carbon dioxide (CO_2_) (Sanyo, Japan) at 37^o^C for 24 h [9]. Following incubation, starter cultures from all bacteria strains were combined and mixed with low-fat milk in a ratio of 1:9 and then incubated at 37°C for another 24 h. The fermented product made the mother culture of PSPY.

### Plant

Purple sweet potatoes (*I. batatas* [L.] Lam) were obtained from a local plantation in Tanjungsari, Sumedang, West Java, Indonesia. It was identified by the Faculty of Agriculture, Universitas Padjadjaran, with breeding varieties registration number: 733/PVHP/2019.

### Experimental animals

Animal experimentation was conducted at the Animal Laboratory of the Department of Biomedical Sciences, Faculty of Medicine, Universitas Padjadjaran. A total of 65 Balb/c mice of both sexes aged 6-8 weeks with a 25-30 g of body weight were obtained from PT. Biofarma Bandung, West Java, Indonesia. The mice were acclimatized for 7 days in a 41.5×29.5×20 cm plastic cage with husk as the base, with five mice per cage. They were kept in a homogenous temperature and humidity with natural lighting.

### Preparation of PSPY

Purple sweet potatoes were washed and steamed, and then 3 g of steamed purple sweet potato was peeled and cut into small pieces. Warm water was added with a 1:1 ratio to the weight of sweet potato and then mashed with a blender to produce puree. Low-fat milk, sweet potato puree, and bacterial starter were mixed with the proportion of 6:3:1. The mixture was then incubated in a 5% CO_2_ incubator at 37°C for 24 h until fermented into a yogurt product [9]. The product of PSPY contains 104 mg/dl of anthocyanin (No.660/Lab.uji-DT/FTIP/SF/2018).

### Acute oral toxicity study

Fifteen female balb/c mice were randomly distributed into three groups evenly. Group 1 served as the control group in which the mice were given distilled water. Groups 2 and 3 were given PSPY with the dosage of 2 and 5 g/kg body weight, respectively. The treatment was administered 1 time peroral (orogastric gavage) and then the mice were observed for 14 days. Observation on each mouse was documented daily to identify signs of toxicity and death. Body weight was measured 3 times; once before administering the PSPY and on the 7^th^ and 14^th^ day of observation. At the end of the study, all mice were sacrificed by euthanasia using intraperitoneal ketamine. Livers and kidneys were extracted and examined for their surface, consistency, and any color change to detect possible organ damage. This study was conducted based on Organization for Economic Co-operation and Development (OECD) guidelines No. 420 about Acute Oral Toxicity–Fixed Dose Procedure [10].

### Sub-chronic oral toxicity study

Fifty mice were randomly divided into five groups consisting of five males and five females in each group. The treatment was administered only once directly into the stomach (orogastric gavage) to ensure an equal dose for every mouse. One control group was treated with distilled water. Three experimental groups received PSPY of 12, 20, and 40 g/kg body weight. The last group was designated as “satellite group” which was treated with 40 g/kg body weight of PSPY and kept for 14 days longer than the other groups to observe reversibility, delayed, or persistence of toxic effect. The Control group and three experimental groups were sacrificed 28 days after PSPY administration, while the satellite group was sacrificed 14 days later than other groups by euthanasia using intraperitoneal ketamine. This study was performed based on OECD guidelines No. 407 and the previous study [11,12].

### Clinical signs and body weight

Animals were observed daily for toxicity signs which are cyanosis, tremor, hypersalivation, piloerection, diarrhea, vomit, and death. They were monitored weekly for body weight from the starting dose until the end of the study. The body weight was measured using ACIS Digital Precision Balance 0.3 kg AD-300i (A&D, Japan). The result was recorded in a gram unit [13-15].

### Relative organ weight

The organ was dried with filter paper before being weighed. The number recorded is the absolute organ weight. Then the weight of mice was divided by absolute organ weight to get the relative organ weight [13,14].

### Necropsy and histological examination

The liver and kidney’s color, surface, and consistency were observed soon after the mice had been sacrificed. The liver was dissected and fixed in 4% paraformaldehyde (P6148, Sigma-Aldrich, Germany). The organs were embedded in paraffin blocks (Sigma-Aldrich) and sectioned into 4 mm thick and then stained with Haematoxylin-Eosin (PATH-MY001, Pathchem). The stained sections were examined under a light microscope (Olympus CX-21, Japan), and any abnormality was analyzed in comparison to the control group [13,14].

### Hematological and biochemical analysis

Whole blood was obtained from the mice through their heart and collected in a potassium EDTA (Becton Dickinson, Franklin Lakes, New Jersey, USA) tube. Hematology profile was analyzed using an automatic hematology analyzer (Sysmex Corp., Japan). Transaminase enzymes alanine aminotransferase (ALT) and aspartate aminotransferase (AST) were analyzed using the Lab kit from Chemelex, S. A. and the results were read at 340 nm wavelength on a spectrophotometer (Infinite 200 Pro, TECAN, Switzerland). The absorbance difference and the average absorbance difference were calculated and multiplied with 1750. Then, the concentration was expressed in units per liter.

### Statistical analysis

Statistical data were evaluated using Statistical Package for the Social Sciences (SPSS) 22.0 (IBM SPSS Statistics, USA). Normal distribution was tested using the Shapiro–Wilk test, and homogeneity was tested using the Lavene test. The data were analyzed using a one-way Analysis of Variance followed by the Dunnett *post hoc* study if it was normally distributed and homogeneity was accepted. Otherwise, the data were analyzed with a non-parametric test, Kruskal–Wallis, and Mann–Whitney. The statistical significance was accepted when p<0.05.

## Results

### Acute oral toxicity study

**Figure-1:**
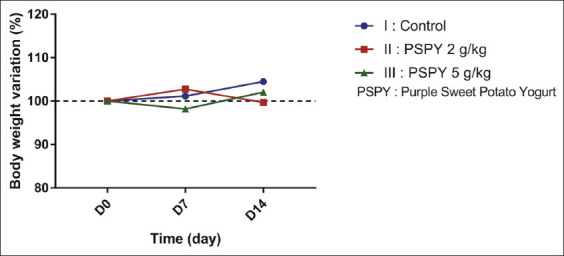
Body weight variation of mice treated with a single dose of PSPY after 14 days. PSPY=Purple sweet potato yogurt.

**Figure-2:**
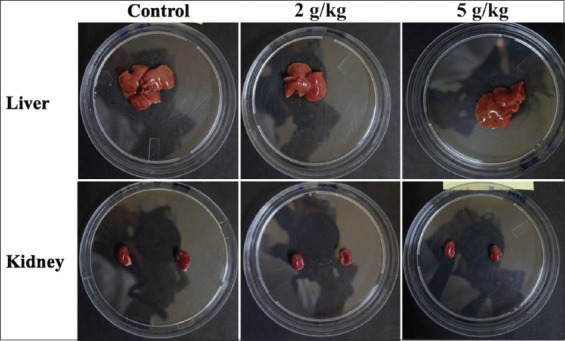
Gross examination of the liver and kidney of mice treated with a single dose of PSPY after 14 days. PSPY=Purple sweet potato yogurt.

After the orogastric gavage administration of PSPY at the beginning of the study, each animal model was observed daily for clinical signs of toxicity and death for 14 days. More attention was given to the first 4 h after administration. Clinical observation showed that consumption of PSPY did not cause death, behavioral changes, and clinical signs of toxicity. No significant difference was observed between the control and treatment groups (Figure-1). Macroscopic examination of the liver and kidneys in this study did not show any abnormalities of color, surface, and consistency (Figure-2). The liver and kidneys’ relative weight did not show any significant difference between all groups (Table-1).

**Table-1:** Relative organ weight of mice treated with a single dose of PSPY after 14 days.

Relative weight	Units	Control	2 g/kg	5 g/kg
Liver	g/100 g	6.22±0.39^NS^	6.11±1.46	5.27±0.43
Kidney	g/100 g	1.24±0.13^NS^	1.22±0.04	1.29±0.09

Results are expressed as a mean±SD. NS=Not significantly different from other groups (p>0.05). PSPY=Purple sweet potato yogurt

### Sub-chronic oral toxicity study

**Figure-3:**
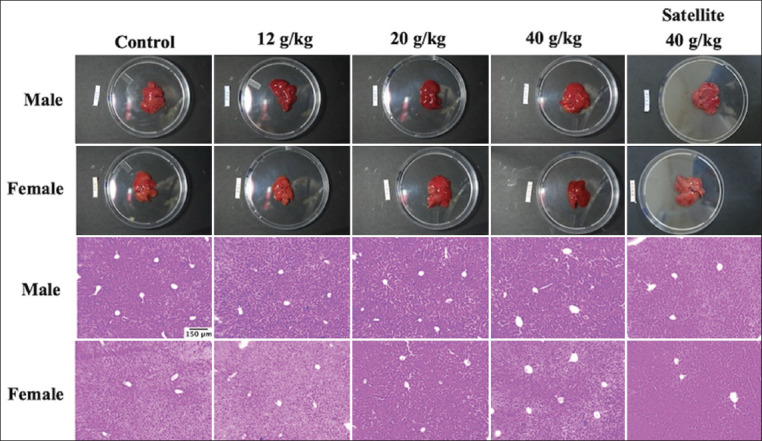
Gross examination and histological findings of the liver after 28-days and satellite group orogastric gavage administration of PSPY (Hematoxylin and eosin, 100×). PSPY=Purple sweet potato yogurt.

No toxicity sign was observed after 28 days of orogastric gavage administration of PSPY as well as the satellite group. There were no statistically significant (p>0.05) differences of relative liver weight among groups presented in Table-2, including the satellite group and no abnormalities of gross examination (color, consistency, and surface) of the liver compared with control groups. Histological findings after 28 days of oral administration of PSPY were normal (Figure-3). There were no statistically significant (p>0.05) differences among liver-associated enzymes (Table-3) and in hematology profiles among groups (Table-4).

**Table-2:** Relative liver weight of mice treated with PSPY for 28 days and satellite groups*.*

Liver weight	Unit	Control	Test groups			Satellites group
	
			12 g/kg/day	20 g/kg/day	40 g/kg/day	40 g/kg/day
Male	g/100 g	6.05±0.45^NS^	5.90±0.72	5.08±0.34	5.87±0.59	5.92±0.61
Female	g/100 g	5.86±0.88^NS^	5.08±0.46	5.32±0.47	5.18±0.82	6.24±0.71

Results are expressed as a mean±SD. NS=Not significantly different from other groups (p>0.05). PSPY=Purple sweet potato yogurt

**Table-3:** ALT and AST of mice treated with PSPY for 28 days and satellite groups.

Parameter	Unit	Control	Test groups			Satellite group
	
			12 g/kg/day	20 g/kg/day	40 g/kg/day	40 g/kg/day
Male						
ALT	U/L	3.30±2.39^NS^	5.61±6.42	6.47±2.87	3.21±1.59	5.20±1.17
AST	U/L	23.45±9.25^NS^	25.42±12.03	24.59±9.60	13.61±8.92	20.41±4.34
Female						
ALT	U/L	6.28±2.40^NS^	12.91±7.59	5.56±1.62	8.98±4.37	9.21±2.92
AST	U/L	22.70±12.89 ^NS^	19.56±8.67	20.90±10.27	21.32±9.54	19.95±4.13

Results are expressed as a mean±SD. NS=Not significantly different from other groups (p>0.05). ALT=Alanine aminotransferase, AST=Aspartate aminotransferase, PSPY=Purple sweet potato yogurt, U/L=Units per liter

**Table-4:** Hematology profile of mice treated with PSPY for 28 days.

Parameters	Control	Test groups		

		12 g/kg/day	20 g/kg/day	40 g/kg/day
Male				
white blood cells (103/μL)	6.27±1.15	5.35±2.20	5.57±1.54	4.55±1.81
Lymphocyte (103/μL)	4.30±1.16	3.59±2.32	3.07±0.94	3.01±1.93
Monocyte (103/μL)	0.92±0.35	1.04±0.29	0.86±0.11	0.80±0.20
Granulocyte (103/μL)	1.08±0.58	0.71±0.24	1.63±1.25	0.73±0.41
Lymphocyte (%)	68.16±12.45	63.12±13.54	55.70±14.96	61.48±19.00
Monocyte (%)	14.51±4.14	22.68±11.55	16.13±3.84	19.64±7.79
Granulocyte (%)	17.30±9.68	14.22±5.58	28.16±17.76	18.90±14.49
RBC (106/μL)	5.41±0.47	5.21±0.53	5.77±0.70	5.02±0.55
HB (g/dl)	12.10±1.46	12.16±1.18	14.53±1.62	11.58±0.32
HCT (%)	24.42±3.77	23.80±1.72	30.03±4.94	22.78±4.94
MCV (fl)	45.00±5.83	45.60±2.60	52.00±4.58	45.40±3.91
MCH (pg)	22.38±2.19	23.32±1.52	25.20±1.08	23.16±2.40
MCHC (g/dl)	49.83±2.46	51.10±1.52	48.66±2.60	50.86±3.69
Platelet (10^3^/μL)	299.50±136.12	406.60±127.02	422.67±130.00	440.80±336.57
Female				
WBC (10^3^/μL)	3.97±1.45	3.34±1.29	6.11±1.21	4.44±2.38
Lymphocyte (10^3^/μL)	2.45±1.22	2.26±0.93	4.65±0.77	2.87±1.73
Monocyte (103/μL)	0.72±0.34	0.67±0.32	0.54±0.38	0.76±0.42
Granulocyte (103/μL)	0.79±0.62	0.41±0.33	0.91±0.64	0.81±0.50
Lymphocyte (%)	61.5±15.9	67.9±10.6	77.2±12.0	64.8±11.6
Monocyte (%)	16.80±5.43	20.50±5.74	8.34±4.93*	16.5±2.17
Granulocyte (%)	21.66±15.32	11.60±5.70	14.44±8.76	18.68±10.57
RBC (106/μL)	4.73±0.91	5.22±0.97	5.49±0.72	5.06±1.69
HB (g/dl)	11.34±2.36	12.26±2.14	13.08±1.54	12.08±4.00
HCT (%)	21.37±4.56	23.71±3.50	26.45±2.19	23.90±7.74
MCV (fl)	45.20±5.21	45.80±4.76	48.60±4.39	47.80±3.49
MCH (pg)	24.04±2.66	23.72±2.71	23.94±2.30	24.14±2.39
MCHC (g/dl)	53.14±1.04	51.62±2.42	49.36±3.27	50.58±1.56
Platelet (10^3^/μL)	285.60±123.54	279.00±125.38	306.20±155.30	249.20±124.65

Results are expressed as a mean±SD. *p<0.05 values are significantly different from control. PSPY=Purple sweet potato yogurt, WBC=White blood cells, RBC=Red blood cells, HB=Hemoglobin, HCT=Hematocrit, MCV=Mean corpuscular volume, MCH=Mean corpuscular hemoglobin, MCHC=Mean corpuscular hemoglobin concentration

## Discussion

In this study, PSPY as a proposed functional food was studied for its acute and sub-chronic toxicity. Purple sweet potato contained a high level of anthocyanin. Anthocyanin has various biological activities such as antioxidant, anti-dyslipidemia, and anti-inflammatory activities [5,6]. The current study showed that PSPY did not exhibit any acute and sub-chronic toxicity after oral administration in mice.

### Acute oral toxicity study

Administration of PSPY to mice for 14 days did not result in any visible signs of toxicity, behavioral changes and death (Figure-1). The results of acute oral toxicity study indicated that the median lethal dose (LD_50_) of this product is higher than 5 g/kg, which might be regarded as safe. This result was supplemented by other studies stating the safety of anthocyanin, which acts as the main bioactive compound in purple sweet potato [16]. However, individual testing of anthocyanin products is still necessary as different plant sources might have different anthocyanin molecules from different plant sources [17].

Data from the gross examination indicated that the PSPY does not have any acute hepatotoxic or nephrotoxic properties (Table-1). Instead, the anthocyanin contained in purple sweet potato might have hepatoprotective effects due to its antioxidant activities, as demonstrated by a previous study [18]. In addition, another study demonstrated that the polysaccharides in purple sweet potato have also exhibited hepatoprotective effects in another study [19]. Another study has found that purple sweet potato anthocyanins can exhibit renal protective effects by reducing inflammation in high-fat diet mice [20]. It also attenuated renal injury by inhibiting inflammatory genes and pro-inflammatory genes [21].

### Sub-chronic oral toxicity study

**Figure-4:**
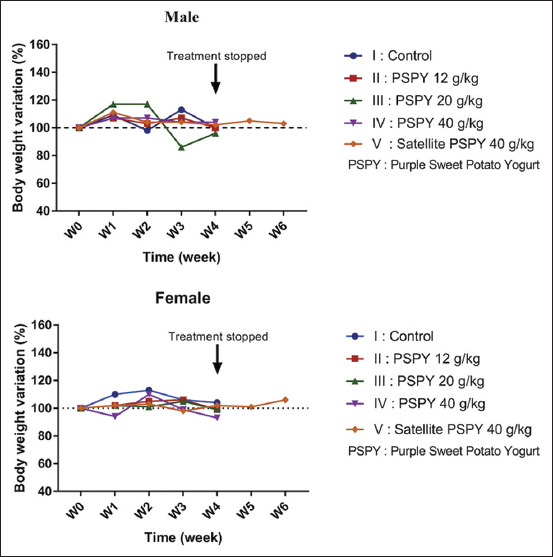
Body weight variation of mice treated with PSPY for 28 days and satellite group. PSPY=Purple sweet potato yogurt.

Administration of PSPY for 28 days showed that there were no toxicity signs during sub-chronic observation. These results were in accordance with the previous study of anthocyanin in bilberry extract for 6 months [16]. The data showed slight changes in the mice’s body weight (Figure-4). Body-weight variation is the first sign of toxicity and a substantial indication of drug and chemical adverse effects in animals [22,23]. In this study, there were no statistically significant differences in body weight among groups, indicating no toxic effect of biochemical substances contained in PSPY. A similar result was obtained from another study that used grape skin extracts (approximately 2.4% anthocyanins by weight) for 90 days and showed no significant differences in body weight [24].

Based on OECD guideline no. 407 [13], stated that targeted organs related to the known properties of the test substance need to be evaluated in more detail. The result of an acute oral toxicity study on the morphology of both kidney and liver showed no toxicity sign manifestation. Therefore, in the sub-chronic toxicity study, the evaluations were focused on the organ that has more involvement in anthocyanin metabolism, which was the liver [25].

Relative liver weight change, either increasing or decreasing, is an essential indicator of organ injury after exposure to toxic substances, increasing or decreasing [22,26]. Hypertrophy of the organ is one of the toxicity indicators [27]. The result of this study demonstrates that there was no statistical significance in relative liver weight (Figure-3). This result is in line with other studies, revealing that repeated oral administration of anthocyanin from grape skin extract for 90 days did not affect relative organ weight [24].

In this study, macroscopic examination of the liver did not show any significant changes, and microscopic examination from histologic observation also supported this finding. Alteration in liver parenchymal cells might be observed when liver transaminase enzymes increase [28]. As stated further, this study revealed that the liver transaminase enzymes in this study were normal. This outcome suggests that the administration of PSPY does not affect liver structure and function.

Liver transaminase enzymes are practical to evaluate liver injury. Since the enzymes are mainly found in cytoplasm, an increase of these enzymes in blood might indicate cell lysis or death [26,29]. This study demonstrates that there was no statistically significant difference in ALT and AST between groups (Table-3). The result indicated that a 28-days oral administration of PSPY in mice did not cause liver injury. Instead, anthocyanin itself has been proven to have a hepatoprotective effect which will reduce ALT and AST in hepatotoxic conditions [22]. In addition, another study also reported that *Bifidobacterium animalis subsp. lactis* and *L. acidophilus* exhibited the highest antioxidant activity, which might prevent liver injury [30].

The hematology profile is sensitive to toxic substances and plays a role as an important index to show physiologic and pathological changes in humans and animals [22]. In this study, there were no statistically significant (p>0.05) differences in hematology profile among groups (Table-4). No alteration in white blood cells, lymphocytes, monocytes, and granulocytes might indicate that the administration of PSPY did not interfere with immune function. There was no alteration in red blood cells, hemoglobin, and hematocrit, indicating that the administration of PSPY did not pose any anemia risk [22,30]. Similar results were also obtained from other studies conducting repeated oral administration of anthocyanin from grape skin extract for 90 days which showed no changes in hematology profile [24].

## Conclusion

This study shows that oral administration of PSPY up to 5 g/kg body weight to mice for 14 days and up to 40 g/kg body weight for 28 days did not result in any clinical signs of toxicity and death, change in body weight, or any sign of liver injury in both studies. In addition, no sign of kidney damage from the acute oral toxicity study was observed. Oral administration of PSPY did not cause acute and sub-chronic oral toxicity. The natural direction is to follow up this study with a toxicity study for a longer-term consumption of PSPY, as it is intended for long-term consumption to improve health. Furthermore, evaluation of other organs that may be affected by the active compound of test material may enrich the data on the safety of PSPY products for mass consumption.

## Authors’ Contributions

AFK: Planned and designed the study and analyzed the data. MYA and PYZ: Equally participated in the collection of the samples and tests. AFK, YP, NO, MYA, PYZ, AA, and WAS: Drafted and revised the manuscript. AFK, WAS, and NA: Data analysis and critical review of the manuscript. All authors read and approved the final manuscript.
